# The comprehensive therapeutic effects of rectal surgery are better in laparoscopy: a systematic review and meta-analysis

**DOI:** 10.18632/oncotarget.14215

**Published:** 2016-12-26

**Authors:** Jiabin Zheng, Xingyu Feng, Zifeng Yang, Weixian Hu, Yuwen Luo, Yong Li

**Affiliations:** ^1^ Department of General Surgery, Guangdong General Hospital and Guangdong Academy of Medical Sciences, Guangzhou, 510080, China; ^2^ Southern Medical University, Guangzhou, 510515, China

**Keywords:** laparoscopy, open, rectal cancer, meta-analysis

## Abstract

**Background:**

Laparoscopic-assisted radical resection of rectal cancer was reported as advantageous compared to laparotomy resection. However, this finding remains controversial, especially given the two recent randomized controlled trials published on The Journal of the American Medical Association (JAMA).

**Objective:**

To perform a meta-analysis that compares the short-term and long-term outcomes of laparoscopic and open surgery for rectal cancer.

**Data source:**

To identify clinical trials comparing laparoscopic and open surgery for rectal cancer published by August 2016, we searched the PubMed, Cochrane Library, Springer Link and Clinicaltrials.gov databases by combining various key words. Data were extracted from every identified study to perform a meta-analysis using the Review Manager 5.3 software.

**Results:**

A total of 43 articles from 38 studies with a total of 13408 patients were included. Although laparoscopic radical rectectomy increased operation time (MD = 37.23, 95% CI: 28.88 to 45.57, *P* < 0.0001), it can significantly decrease the blood loss (MD = –143.13, 95% CI: –183.48 to –102.78, *P* < 0.0001), time to first bowel movement (MD = –0.97, 95% CI: –1.35 to –0.59, *P* < 0.0001), length of hospital stay (MD = –2.40, 95% CI: –3.10 to –1.70, *P* < 0.0001), postoperative complications (OR = 0.78, 95% CI: 0.72 to 0.86, *P* < 0.0001), mortality (OR = 0.40, 95% CI: 0.28 to 0.57, *P* < 0.0001) and the CRM positive rate (OR = 0.64, 95% CI: 0.55 to 0.75, *P* < 0.0001). No significant difference were noted between the groups regarding intraoperative complications, TME completeness and harvesting of lymph nodes. Regarding the long-term survival data, the laparoscopic group was not inferior to laparotomy. Some pooled data, such as 3-year DFS, 5-year OS and 5-year local recurrence were even superior for the laparoscopic group.

**Conclusions:**

Given the definite benefits in short-term outcomes and trending benefits in long-term outcomes that were observed, we recommend laparoscopic surgery be used for rectal cancer resection.

## INTRODUCTION

Rectal cancer is one of the most common malignant tumors. Surgical therapy plays an important role in the comprehensive treatment of rectal cancer, especially when the total mesorectal excision (TME) theory is applied to radical rectal resection, which can significantly improve the prognosis of patients with rectal cancer [1]. With the development of minimally invasive surgery, laparoscopic-assisted radical rectectomy has been accepted by more and more surgeons. Many researches have shown its advantages on postoperative recovery and complications, while some have shown its disadvantages on operation time. The most controversial issues focus on pathology and long-term survival [2]. Therefore, some meta-analyses focused on either short-term or long-term indexes were previously carried out and have tried to solve these problems. Most of these meta-analyses show no significant differences between laparoscopy and open groups [3]. However, two randomized controlled trials (RCTs) published in Journal of American Medical Association (JAMA) were diametrically opposed to the results of previous clinical trials [4, 5]. These two trials concluded that the non-inferiority of laparoscopic compared to open surgery was not established. Therefore, a systematic review and meta-analysis was undertaken to update these studies and evaluate both the short-term and long-term results of laparoscopic and open radical rectectomy.

## RESULTS

### Studies included

**Figure 1:**
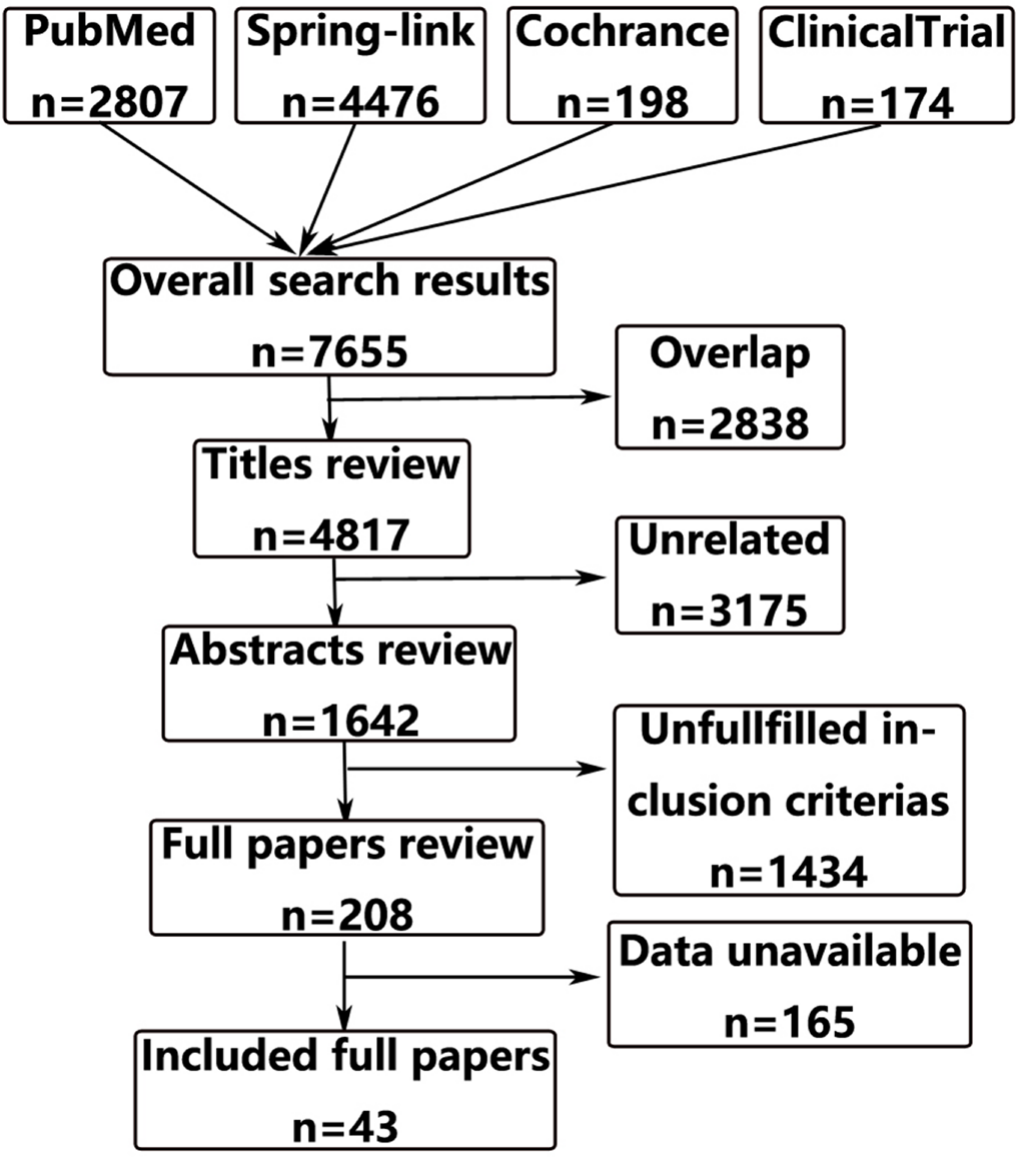
Flow diagram of articles included and excluded

A total of 7655 citations from four databases met our search criteria up to August 2016. Review of the full-text articles revealed 43 articles that adequately matched the inclusion and exclusion criteria, which contained short-term and long-term results from 38 different studies [4–46]. The studies that were included in this meta-analysis included thirteen RCTs and, twenty-five non-RCTs with a total number of 13408 patients who suffered from rectal cancer. The study screening and selection processes are presented in Figure 1. Quality assessment of the included articles and the characteristics of the included patients are presented in Table 1.

**Table 1:** Study characteristics of included studies

Study	Study type	Country	Study period	Number of patients		Score	Conversion rate
				Laparoscopy	Open		
Zhou Z.G.2004	RCT	China	Jun 2001 to Sep 2002	82	89	MR	NR
Pechlivanides G.2007	RCT	Greece	NR	34	39	LR	3%
Braga M.2007	RCT	Italy	NR	83	85	LR	7.2%
Ng S.S.2008	RCT	Hong-Kong	Jul 1994 to Feb 2005	51	48	LR	9.8%
Lujan J.2009	RCT	Spain	Jan 2002 to Feb 2007	101	103	LR	7.9%
Liang X.2011	RCT	China	May 2004 to Apr 2008	169	174	MR	0.6%
Gong J.2012	RCT	China	Sep 2008 to Jul 2011	67	71	MR	2.99%
CLASICC 2013	RCT	UK	Jul 1996 to Jul 2002	253	128	LR	32.4%
COREAN 2014	RCT	Korea	Apr 2006 to Aug 2009	170	170	LR	1.2%
Ng S.S.2014	RCT	Hong-Kong	Aug 2001 to Aug 2007	40	40	LR	7.5%
COLOR II 2015	RCT	Europe	Jan 2004 to May 2010	699	345	LR	17%
ALaKa 2015	RCT	Australasia	Mar 2010 to Nov 2014	238	237	LR	9%
Z6051 2015	RCT	US & Canada	Oct 2008 to Sep 2013	240	222	LR	11%
Leung K.L.2000	Non-RCT	Hong-Kong	Jan 1993 to Jan 1996	25	34	9	8.0%
Anthuber M.2002	Non-RCT	Germany	Jan 1996 to Mar 2003	101	334	9	0
Feliciotti F.2003	Non-RCT	Italy	May 1992 to Apr 2002	52	34	9	12.3%
Wu W.X.2004	Non-RCT	China	Apr 2002 to May 2003	18	18	8	0
Breukink S.O.2005	Non-RCT	Netherlands	Apr 1996 to Mar 2003	41	41	8	9.8%
Morino M.2005	Non-RCT	Italy	Apr 1994 to Apr 2002	98	93	9	18.4%
Law W.L.2006	Non-RCT	Hong-Kong	Jun 2000 to Dec 2004	98	167	8	12.2%
Lelong B. 2007	Non-RCT	France	Jan 1998 to Oct 2004	104	68	8	14.4%
Veenhof A.A.2007	Non-RCT	Netherlands	Feb 1999 to Nov 2005	50	50	9	8.0%
Ströhlein M.A.2008	Non-RCT	Germany	1998 to 2005	114	275	9	21.9%
González Q.H.2009	Non-RCT	Mexico	Nov 2005 to Nov 2007	28	28	8	0
Gouvas N.2009	Non-RCT	Greece	Jan 1998 to Mar 2007	45	43	8	9%
Khaikin M.2009	Non-RCT	America	Nov 2004 to Jul 2006	32	50	9	12.5%
Koulas S.G.2009	Non-RCT	Greece	Oct 1998 to Dec 2006	57	60	7	7.0%
Laurent C.2009	Non-RCT	France	1994–2006	238	233	9	15.1%
Baik S.H.2011	Non-RCT	Korea	Sep 2001 to Sep 2005	54	108	9	11.1%
McKay G.D.2011	Non-RCT	Australia	Jan 2001 to Dec 2008	157	388	8	8.3%
Gunka I.2012	Non-RCT	Czech	Jan 2001 to Dec 2006	75	70	8	5.9%
Jefferies M.T.2012	Non-RCT	UK	Feb 2007 to Jun 2010	16	25	8	12.5%
Kellokumpu I.H.2012	Non-RCT	Finland	1999 to 2006	100	91	9	22%
Seshadri R.A.2012	Non-RCT	India	Jan 2004 to Jan 2010	72	72	9	4.2%
Lujan J.2013	Non-RCT	Spain	2006 to Jul 2013	1387	3018	9	17.4%
Cho M.S.2015	Non-RCT	Korea	Jan 2003 to Jun 2008	211	422	9	3%
Kim J.H.2015	Non-RCT	Korea	Jan 2002 to Dec 2011	131	176	7	NR

Abbreviations: RCT: randomized controlled trial; LR: low risk; MR: moderate risk; NR: no reported.

### Surgical outcomes

**Figure 2:**
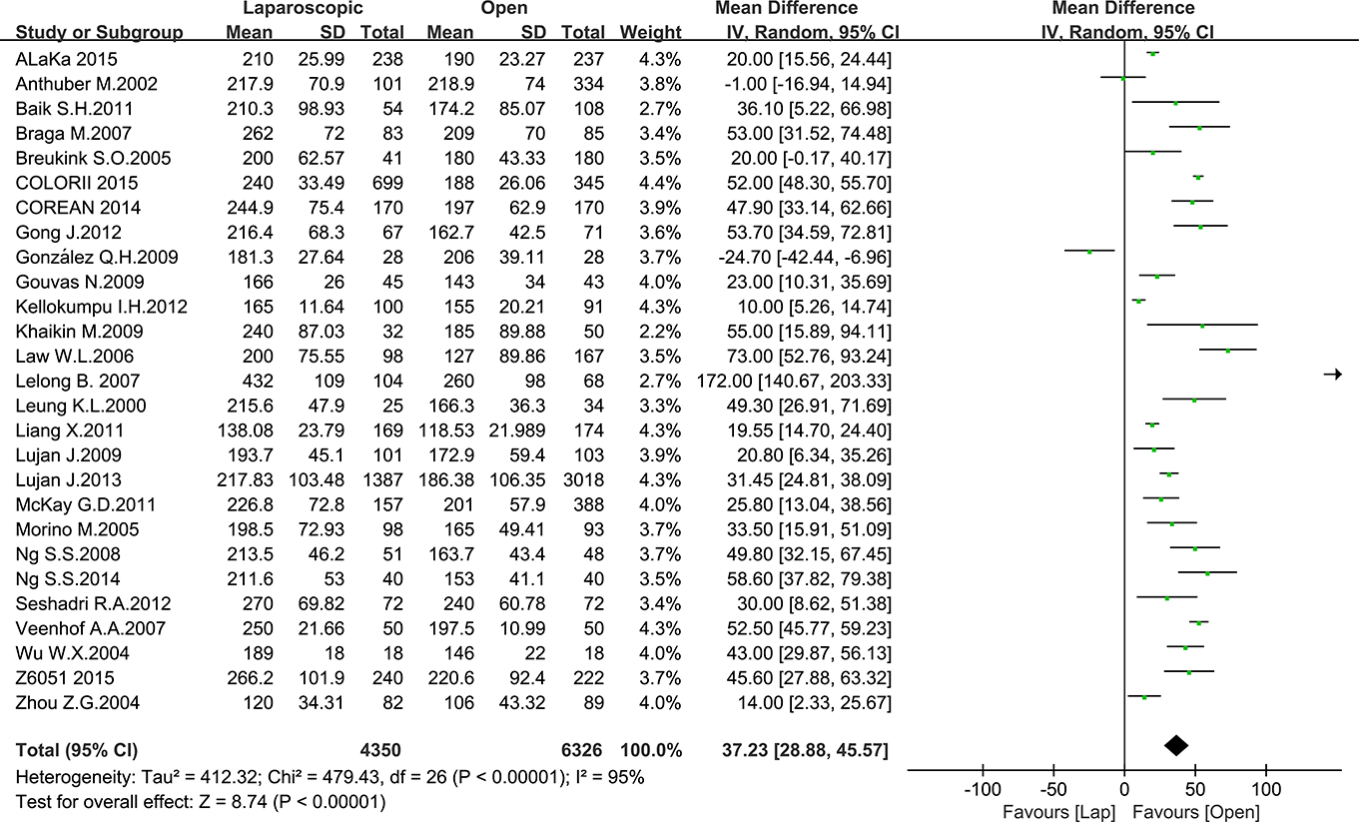
Pooled MD for operation time of including studies

Operation time, blood loss and intraoperative complications are the most important outcomes for surgical procedure. Operation time was reported in 18 studies, including 4350 patients in the laparoscopic group and 6326 patients in the open group. The mean operation time ranged from 138 to 266 min in the laparoscopic group and from 127 to 240 min in the open group. Significantly longer operation times were noted in the laparoscopic group compared to the open group (MD = 37.23, 95% CI: 28.88 to 45.57, *P* < 0.0001, I^2^ = 95%) (Figure 2).

**Figure 3:**
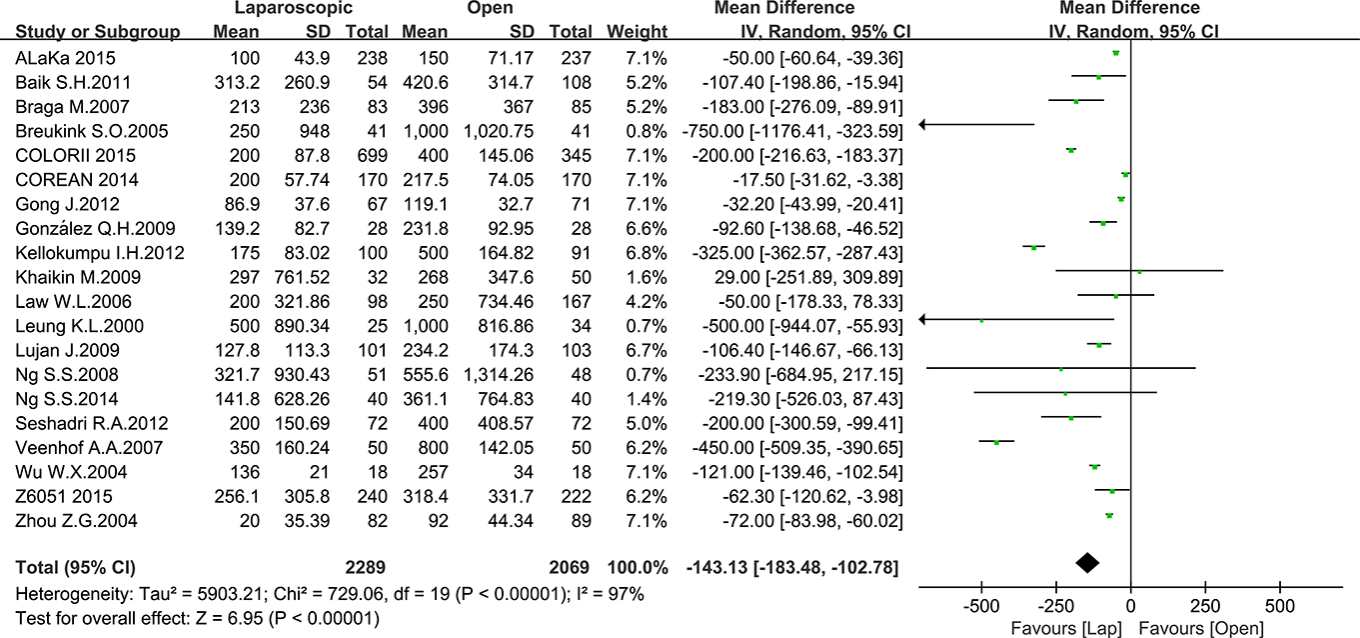
Pooled MD for blood loss of including studies

There were also 18 studies that reported operative blood loss, with a total number of 2289 patients in the laparoscopic group and 2069 patients in the open group. The mean blood loss ranged from 20 to 500 ml and 92 to 1000 ml for the laparoscopic and open groups, respectively. In this meta-analysis, a significant difference was noted between the laparoscopic and open groups. The blood loss in the laparoscopic group was obviously less than the open group (MD = –143.13, 95% CI: –183.48 to –102.78, *P* < 0.0001, I^2^ = 97%) (Figure 3) Given that the I^2^ was greater than 50%, the random-effect model was used to calculate the pooled MD for operation time and blood loss.

**Figure 4:**
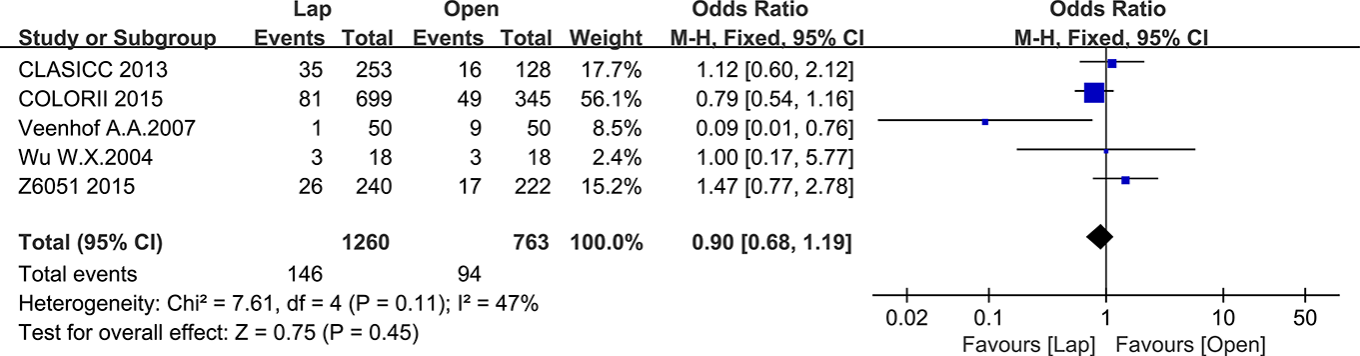
Pooled OR for intraoperative complications of including studies

Five studies reported intraoperative complications, including 1260 patients in the laparoscopic group and 763 patients in the open group. No significant differences were noted in intraoperative complications rate between these two groups (OR = 0.90, 95% CI: 0.68 to 1.19, *P* = 0.45, I^2^ = 47%) (Figure 4).

### Postoperative outcomes

**Figure 5:**
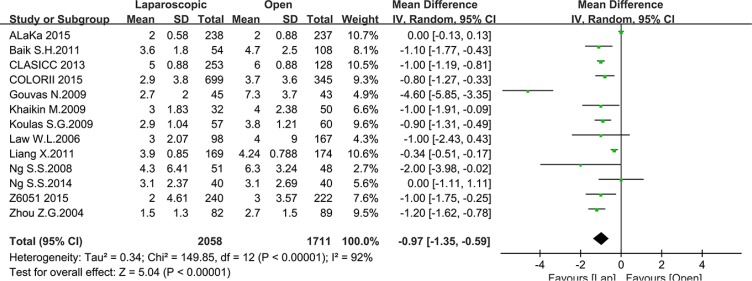
Pooled MD for first bowel movement of including studies

**Figure 6:**
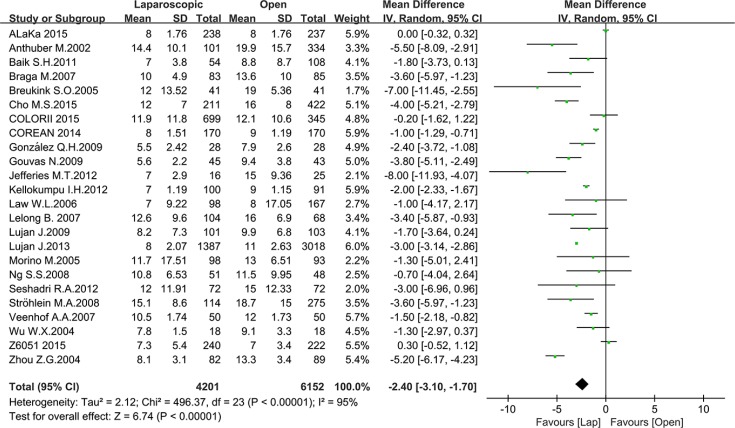
Pooled MD for length of hospital stay of including studies

Postoperative outcomes refer to recovery speed and postoperative complications. We included the time to first bowel movement, length of hospital stay, total postoperative complications and postoperative mortality in our analysis. Thirteen studies reported the time to first bowel movement with a total number of 3769 patients for meta-analysis. As shown in Figure 5, the time to first bowel movement was shorter in the laparoscopic group than in the open group (MD = –0.97, 95% CI: –1.35 to –0.59, *P* < 0.0001, I^2^ = 92%). Twenty-four studies reported the time of hospital stay with a total number of 10353 patients for meta-analysis. In this meta-analysis, a significant difference was noted between the laparoscopic group and open group (MD = –2.40, 95% CI: –3.10 to –1.70, *P* < 0.0001, I^2^ = 95%) (Figure 6). Given that the I^2^ was greater than 50%, the random-effect model was used to calculate the pooled MD for the time to first bowel movement and length of hospital stay.

**Figure 7:**
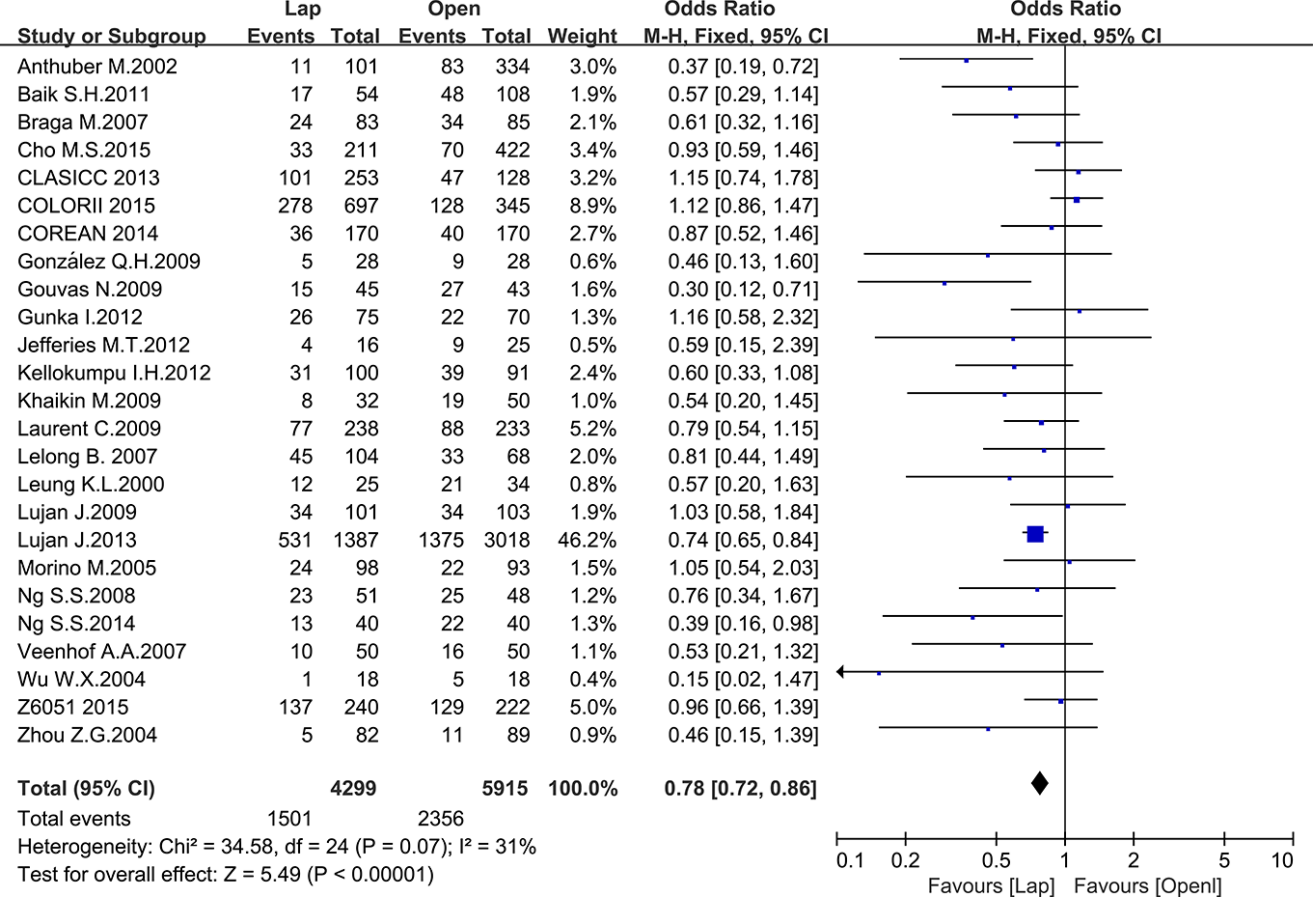
Pooled OR for postoperative complications of including studies

**Figure 8:**
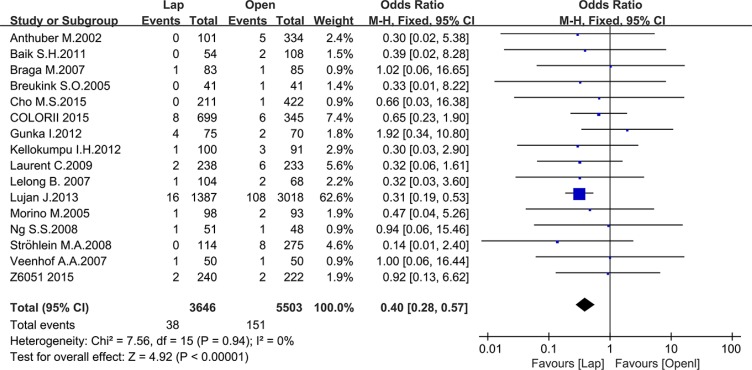
Pooled OR for postoperative mortality of including studies

There were 17 studies that reported postoperative complications and 16 studies that reported mortality, including 10214 and 9149 patients, respectively. The meta-analysis showed fewer postoperative complications (OR = 0.78, 95% CI: 0.72 to 0.86, *P* < 0.0001, I^2^ = 31%) (Figure 7) and lower mortality in the laparoscopic group (OR = 0.40, 95% CI: 0.28 to 0.57, *P* < 0.0001, I^2^ = 0%) (Figure 8).

### Pathology outcomes

**Figure 9:**
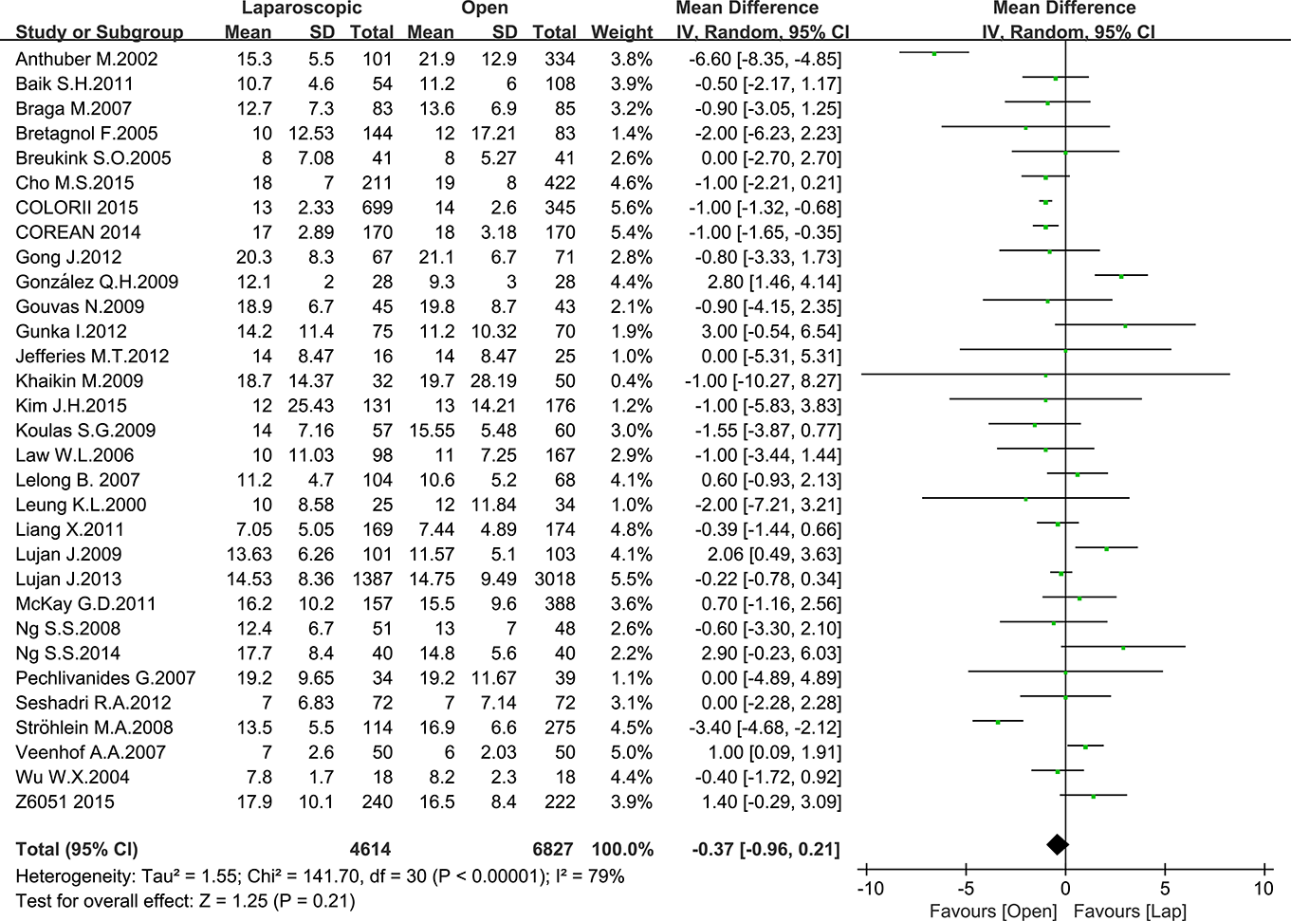
Pooled MD for number of harvested lymph nodes of including studies

**Figure 10:**
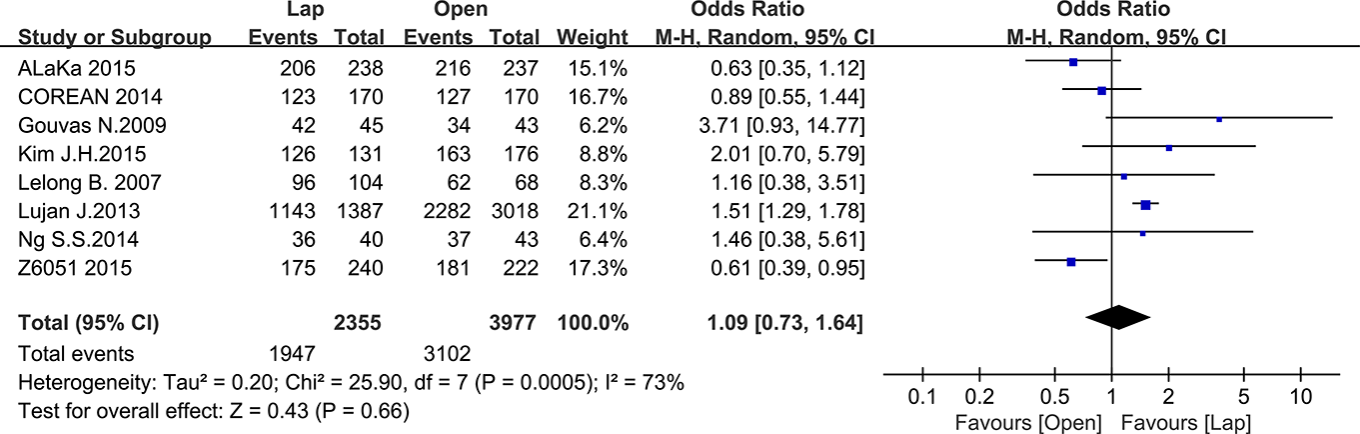
Pooled OR for TME completeness of including studies

Pathology outcomes include lymph nodes harvesting, circumferential resection margin (CRM) positive rate and completeness of the TME. Most of the studies reported the number of harvested lymph nodes, with 10935 patients for meta-analysis. As Figure 8 shown, there was no significant difference between the laparoscopic and open groups in the number of harvested lymph nodes (MD = –0.37, 95% CI: –0.96 to 0.21, *P* = 0.21, I^2^ = 79%) (Figure 9). additionally, there was no significant difference in completeness of the TME between the two groups according to the meta-analysis of eight studies (OR = 1.09, 95% CI: 0.73 to 1.64, *P* = 0.66, I^2^ = 73%) (Figure 10). Given that significant heterogeneity existed, the random-effect model was applied to these two analyses.

**Figure 11:**
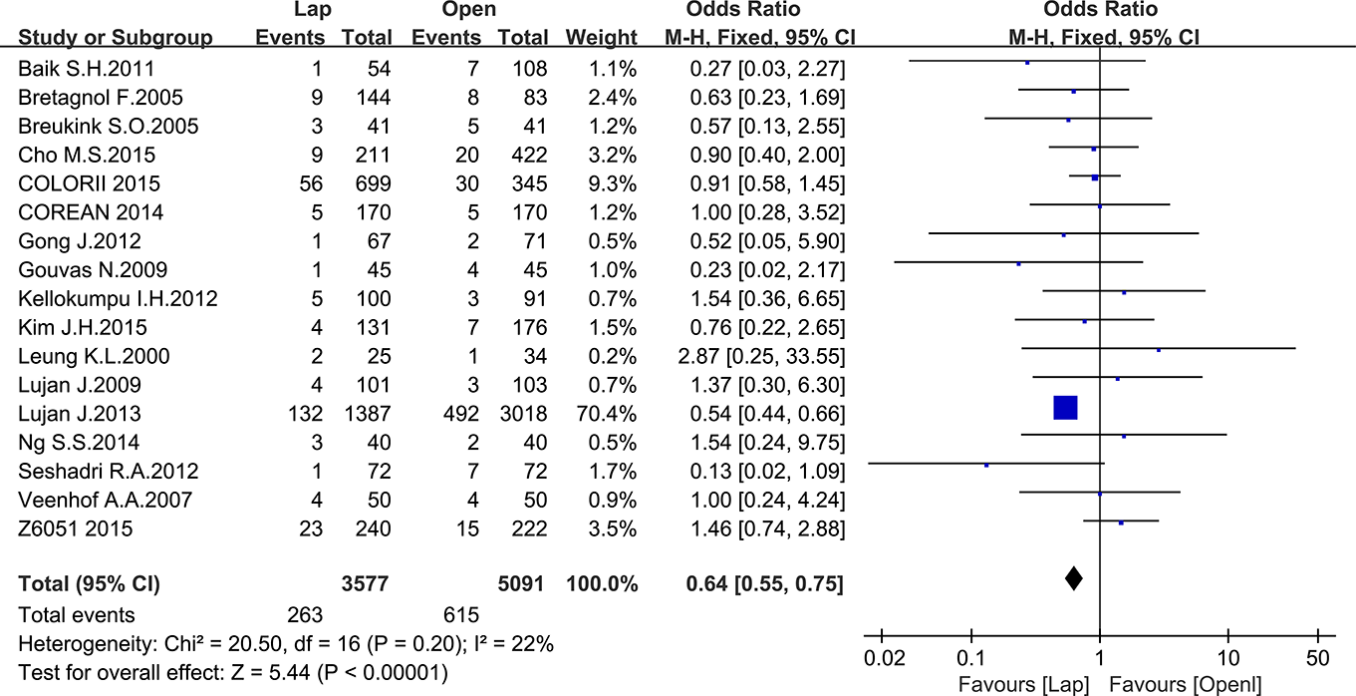
Pooled OR for CMR positive rate of including studies

Seventeen studies reported the CRM positive rate, including 3577 patients in the laparoscopic group and 5091 patients in the open group. Meta-analysis showed a significantly lower CRM positive rate in the laparoscopic group (OR = 0.64, 95% CI: 0.55 to 0.75, *P* < 0.0001, I^2^ = 22%) (Figure 11).

### Long-term survival data

**Figure 12:**
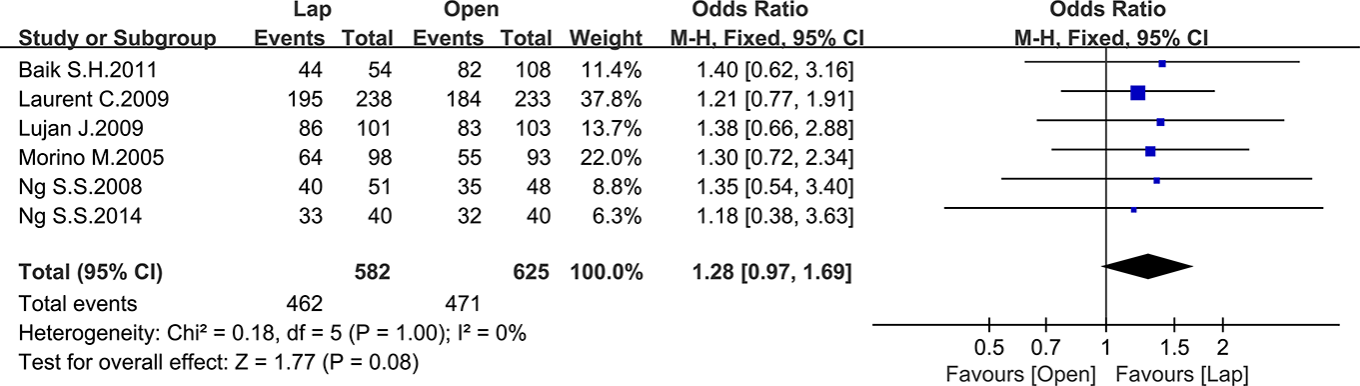
Pooled OR for 5-year DFS of including studies

**Figure 13:**
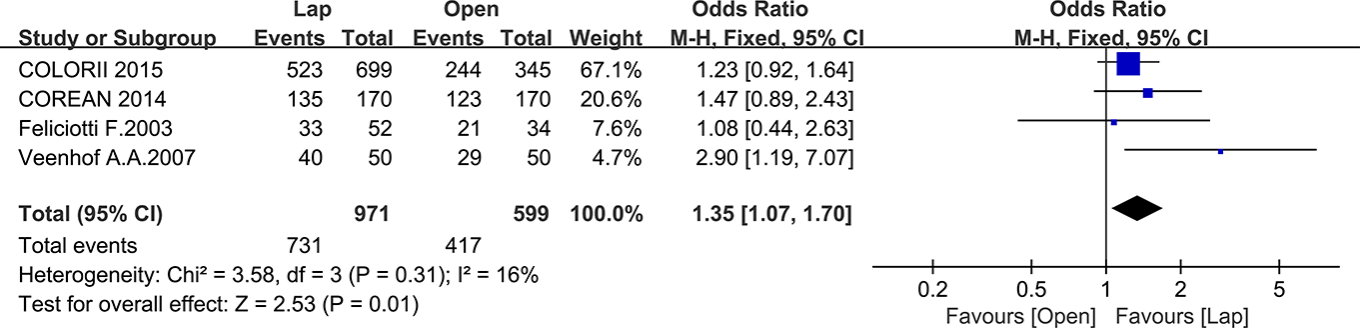
Pooled OR for 3-year DFS of including studies

Ten studies reported the disease-free survival (DFS), 4 of which reported the 3-year DFS while the others reported the 5-year DFS. There was no significant difference between the laparoscopic and open group in 5-year DFS (OR = 1.28, 95% CI: 0.97 to 1.69, *P* = 0.08, I^2^ = 0%) (Figure 12), whereas the laparoscopic group showed a significantly higher 3-year DFS compared to the open group (OR = 1.35, 95% CI: 1.07 to 1.70, *P* = 0.01, I^2^ = 16%) (Figure 13).

**Figure 14:**
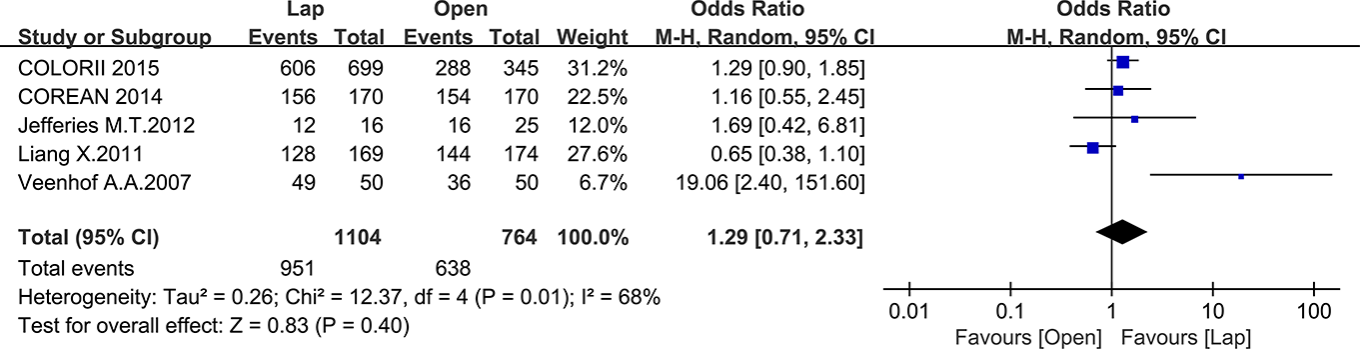
Pooled OR for 3-year OS of including studies

**Figure 15:**
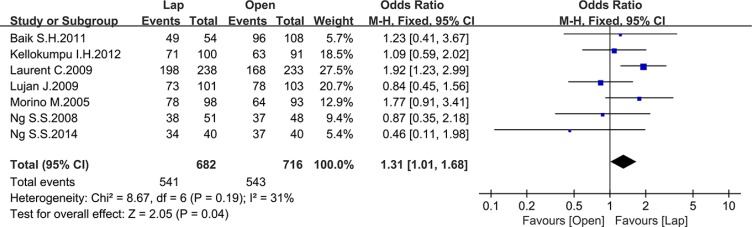
Pooled OR for 5-year OS of including studies

Twelve studies reported the overall survival (OS), 5 of which reported the 3-year OS while the others reported the 5-year OS. There was no significant difference between the laparoscopic and open groups in 3-year OS (OR = 1.29, 95% CI: 0.71 to 2.33, *P* = 0.40, I^2^ = 68%) (Figure 14), whereas the laparoscopic group showed a significant higher 5-year OS compared to the open group (OR = 1.31, 95% CI: 1.01 to 1.68, *P* = 0.04, I^2^ = 31%) (Figure 15).

**Figure 16:**
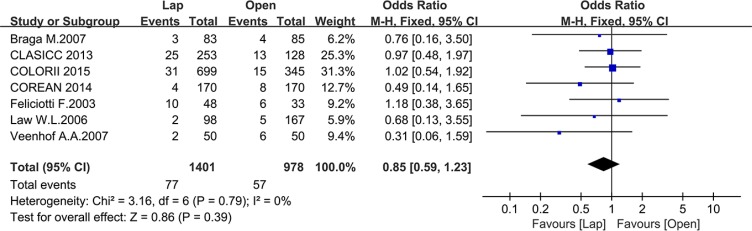
Pooled OR for 3-year local recurrence rate of including studies

**Figure 17:**
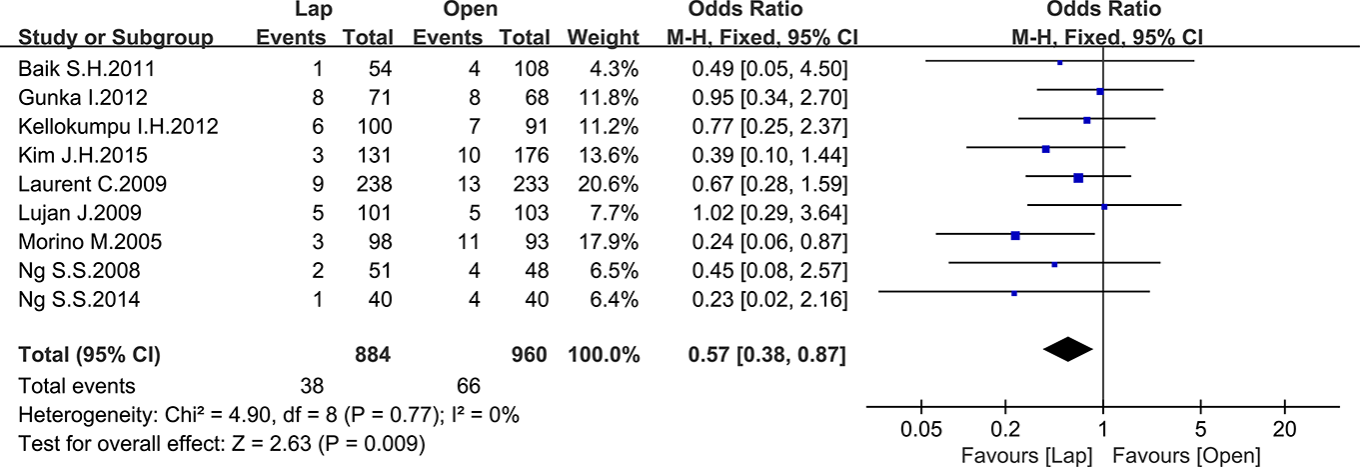
Pooled OR for 5-year local recurrence rate of including studies

Seven studies reported the 3-year local recurrence (LR) and nine studies reported the 5-year LR. There was no significant difference in the 3-year LR between the two groups (OR = 0.85, 95% CI: 0.59 to 1.23, *P* = 0.39, I^2^ = 0%) (Figure 16), while the laparoscopic group showed a lower 5-year LR (OR = 0.57, 95% CI: 0.38 to 0.87, *P* = 0.009, I^2^ = 0%) (Figure 17).

## DISCUSSION

Laparoscopic-assisted radical rectectomy has been widely applied in clinical practice since it was first reported. However, the efficacy and safety of this procedure are still controversial. Many clinical trials and meta-analyses have been designed to prove its feasibility. When everything seemed to reach an agreement that laparoscopic-assisted radical rectectomy is feasible, two randomized clinical trials published in JAMA went against this opinion [4, 5]. Therefore, we conducted this meta-analysis to pool the latest research data and probe these discrepancies. Because too many outcomes were reported, we choose some of the most important outcomes for analysis and divided them into four types, including surgical outcomes, postoperative outcomes, pathology outcomes and long-term survival data.

We choose three surgical and postoperative outcomes for meta-analysis, which are the important indicators of surgical safety. Given the irregularity of laparoscopic skill levels, the observed heterogeneity in surgical outcomes was expected. We had to analyze the data using a random-effect model. As was shown in the meta-analysis, the operation time was significantly longer in laparoscopic group. After all, laparoscopic radical rectectomy is a relatively new surgical approach in comparison to open surgery. Most surgeons lack proficiency in laparoscopic surgery. And this is why many people still have doubts about laparoscopic surgery. As practice and proficiency increase, the operation time will undoubtedly decrease.

On the other hand, laparoscopy has its own advantages. It can magnify the operative region and help to identify and protect the important structures, which makes the operation more precise. What is more, it decreases the disruption of normal tissue. As a result, the blood loss, complication and mortality rates were lower, and the time to first bowel movement and length of hospital stay were shorter, which indicates less over tissue damage and faster recover.

Pathology outcomes were used as the main outcomes in the latest two JAMA trials. The quality of the specimen closely relates to the degree of radical resection. TME completeness, CRM positive rate and number of harvested lymph nodes were the most important indicators. In our meta-analysis, the CRM positive rate was significantly lower in the laparoscopic group, whereas no significant differences in harvested lymph nodes numbers or TME completeness was seen. In our opinion, however, pathology outcomes should be treated as important indicators of surgical quality but not as the endpoint of cancer therapy. The main evaluation criteria of surgical approaches should be based on patients’ long-term survival benefits, especially the OS and DFS. For example, the Dutch trial, with a 15-year follow-up period, meets this criteria quite well [47]. As demonstrated in the COREAN trial [46] and COLOR II trial [11], some short-term outcomes, including pathology outcomes, in the open group were superior to those in the laparoscopic group, but all these advantages did not transform into a survival benefit.

In this meta-analysis, the pooled long-term survival data demonstrated that laparoscopic surgery was not inferior to laparotomy. The 3-year DFS, 5-year OS and 5-year local recurrence rates were significantly superior in patients who received laparoscopic resection. This result is reasonable given the advantages mentioned above. However, some survival data still did not reach statistical significance in this meta-analysis. As far as we are concerned, the laparoscopic radical rectectomy may not show its advantage in long-term survival given our collective history of unskilled laparoscopic technique. The prognosis of patients with rectal cancer will significantly improve as laparoscopy technique becomes more mature. We are looking forward to the survival data of the latest trials and even more reasonably designed new trials.

In conclusion, given the definite benefits of short-term outcomes and trending benefits of long-term outcomes that were observed, we recommend laparoscopic surgery be used in rectal cancer resection.

## MATERIALS AND METHODS

### Study selection

A systematical search of all relevant literature published until August 2016 was performed using the following 4 online databases: PubMed, Cochrane Library, Springer Link and Clinicaltrials.gov. The key search terms used in various combinations included “rectal cancer,” “rectal neoplasm,” and “laparoscopy,” “laparoscopic,” “minimally invasive”. All searches and literature selections were independently conducted by two investigators (Zheng and Feng).

### Inclusion and exclusion criteria

All clinical trials that compared laparoscopic surgery and open surgery were included. All literature that failed to fulfill the following criteria were excluded: (1) the patients presented with rectal cancer, (2) the study compared patients who underwent laparoscopy to patients who underwent open surgery (3) the study was written in English. All included studies were independently assessed by two authors (Zheng and Feng).

### Quality assessment

Study quality was assessed independently by two authors (Zheng and Feng). All of the RCTs were assessed by the Cochrane Collaboration's tool, whereas the non-RCT studies were assessed by the Newcastle-Ottawa Scale (NOS). If controversy existed between the two independent evaluations, all of the authors participated in a discussion to resolve the issue. A score above 6 for non-RCT studies indicated high quality, otherwise, a lower score indicated poor quality.

### Data extraction

Data form shortlisted articles were extracted independently by the two authors (Zheng and Feng) and entered into a pre-designed form after reaching a consensus. The main data reported included study characteristics and outcomes. Study characteristics, including the time, country, study type, number of patients, treatment, outcomes and quality score, are presented in Table 1. The study outcomes included operation time, blood loss, harvesting of lymph nodes, recovery time, hospital stay duration, permanent stomas rate, postoperative complications and postoperative death, et al.

### Statistical analysis

Review Manager (RevMan) v5.3 (Cochrane Library) software was used to perform the meta-analysis. The odds ratio (OR) and Mean Difference (MD) were used to analyze the dichotomous data and the continuous data, respectively. For some studies that did not report the mean and standard deviation (SD), we use the method reported by Hozo S.P. et al. [48] to calculate the mean and SD. I^2^ and *Q* tests were used to determine statistical heterogeneity. Fixed-effect models were used in the analyses if *P*-values were greater than 0.1 and the I^2^ was less than 50%. Otherwise, random-effect models were used. In addition, an I^2^ value of less than 25% was defined as low heterogeneity, a value between 25 and 50% was defined as moderate heterogeneity, and a value of I^2^ > 50% was defined as high heterogeneity. *P*-values less than 0.05 were considered significant.
